# 3D vascular mapping reveals multi-organ injury following severe acute pancreatitis

**DOI:** 10.7150/thno.141299

**Published:** 2026-08-24

**Authors:** Xiaomei Liu, Jingtan Zhu, Qiao Shi, Jianyi Xu, Zhang Liu, Yujia Kang, Kunxing Liu, Yuening He, Qihang Yang, Tingting Yu, Dan Zhu

**Affiliations:** 1 MOE Key Laboratory for Biomedical Photonics, Wuhan National Laboratory for Optoelectronics-Advanced Biomedical Imaging Facility, Huazhong University of Science and Technology, Wuhan, Hubei 430074, China.; 2 Optics Valley Laboratory, Wuhan, Hubei 430074, China.; 3 Center for Medical Genetics, School of Life Sciences, MOE Key Laboratory of Rare Pediatric Diseases, Hunan Key Laboratory of Animal Models for Human Diseases, Central South University, Changsha, Hunan 410031, China.; 4 Department of Hepatobiliary and Pancreatic Surgery, Renmin Hospital of Wuhan University, Wuhan, Hubei 430060, China.

**Keywords:** severe acute pancreatitis, 3D vascular imaging, tissue clearing, systemic vascular injury, multi-organ dysfunction

## Abstract

**Rationale:**

Severe acute pancreatitis (SAP) is a life-threatening disease with substantial mortality, primarily resulting from pancreatic microcirculatory failure that initiates multiple organ dysfunction syndrome (MODS). While early microvascular injury is widely recognized as a critical determinant of disease progression, the precise spatiotemporal dynamics and mechanisms underlying vascular damage remain largely elusive, limiting the development of effective therapeutic interventions.

**Methods:**

An integrated imaging method that combines vascular labeling, tissue clearing, and light-sheet microscopy is proposed to realize 3D mapping and quantitative analysis of multi-organ vascular networks in a mouse model of SAP.

**Results:**

We found that SAP induced systemic microvascular disruption accompanied by organ-specific perfusion deficits across multiple vital organs. As the primary site of injury, the pancreas exhibited the earliest and most severe hypoperfusion, whereas remote organs showed a delayed decline in perfusion, with different trends among organs. Specifically, perfusion in the lung, heart, and kidney decreased gradually with disease progression, perfusion in the liver and spleen remained stable within the first 6 hours and then decreased rapidly, while the brain perfusion showed a sharp decrease at the end stage. These distinct perfusion trajectories are consistent with sequential compensatory and decompensatory responses. More importantly, the administration of heparin sodium within 12 hours after SAP induction significantly improved survival, reversed multi-organ hypoperfusion, and preserved microvascular integrity across affected organs.

**Conclusions:**

This study identifies systemic vascular injury as a key driver of multiple organ dysfunction in SAP and provides a convincing evidence that early intervention with heparin sodium can improve multi-organ hypoperfusion, preserve microvascular integrity, and enhance survival.

## Introduction

Acute pancreatitis (AP) is a common pancreatic disorder and a major contributor to gastrointestinal hospitalizations. The incidence was about 0.0337% per year 1, 2, and has steadily increased worldwide over recent decades 2-5. The 20% will develop into severe acute pancreatitis (SAP). SAP is a fatal disease due to substantial local and systemic complications, causing increased morbidity, mortality, and healthcare costs 6-8. Persistent organ failure is associated with a mortality rate exceeding 40% 1.

The injury to the intestine, kidney, lung, liver, and heart in SAP has been reported extensively 9-13. Nevertheless, most studies conducted to date have focused on individual organs or specific time intervals. More and more evidence indicates that vascular injury is an important pathological process in SAP, which causes multi-organ dysfunction by endothelial injury, hemorheological disorders, enhanced capillary permeability, and micro-thrombosis 14-16. There is substantial clinical evidence of vascular injury in AP: a large cohort study of 3,048 subjects showed vascular complications in 26.5% of the cohort, including splanchnic vein thrombosis, portal hypertension, and arterial injury 17. Protection of endothelial tight junctions reduces capillary leakage in SAP models, reinforcing the central role of endothelial dysfunction in SAP pathogenesis 18.

Nevertheless, it remains unknown whether vascular damage takes place simultaneously in all organs or it occurs in an organ-specific order. Traditional histology offers some fragmented two-dimensional images, which do not provide sufficient information to depict the complexity of 3D vascular networks. It is well known that the vascular system is a complex, hierarchical 3D network spanning from large vessels to capillaries in all parts of the body.

During the past years, advanced optical imaging techniques provide important tools for visualizing blood vessels 19, 20, but the imaging depth suffers from the turbid tissue 21. Recently, various tissue optical clearing methods have been proposed 22-27, when combined with fluorescence labeling and light-sheet microscopy, which enable mapping the entire-organ vascular architecture with exceptional spatiotemporal details.

However, applying these tools to SAP will require tailoring labeling and clearing methods to make them compatible with diverse tissue types. Vascular labeling currently relies largely on passive diffusion or direct injection of fluorescent dyes 28-32, which can result in incomplete labeling, low signal strength, or incompatibility with clearing methods 30, 33, 34. Fluorescent perfusion improves vessel labeling of all sizes, but substantial challenges, such as dye extravasation, vascular occlusion, and organ-to-organ variability still remain largely unresolved 35-38. Tissue optical clearing methods include hydrophobic, hydrophilic, and hydrogel approaches 39-41, each offering distinct trade-offs with respect to optical transparency, structural preservation, and fluorescence compatibility 42-44.

In this work, we developed an integrated 3D imaging pipeline to study multi-organ vascular injury in SAP on a systematic basis. We introduced an effective pan-organ vessel-labeling method, WGA-GEL, that provides uniform vascular staining across tissues. By carefully assessing multiple tissue-clearing strategies, we chose SOLID 45 as the best suited to achieve high transparency and low structural deformation. And then we applied light-sheet microscopy to obtain vascular network of organs at different SAP stages. In addition, we administered heparin sodium at different times after SAP induction and measured the organ recovery in order to define a critical therapeutic window.

This study provides a multi-organ view of vascular injury in the course of progression of SAP, shows organ-specific vulnerability and time trends, and presents an early therapeutic window that may alleviate multi-organ dysfunction. These findings advance mechanistic understanding of multi-organ vascular injury in SAP and are useful as a valuable resource for evaluating treatment efficacy and ideal timing, and guiding future translational research.

## Results

### Progression of SAP and the associated multi-organ vascular injury

**Figure 1:**
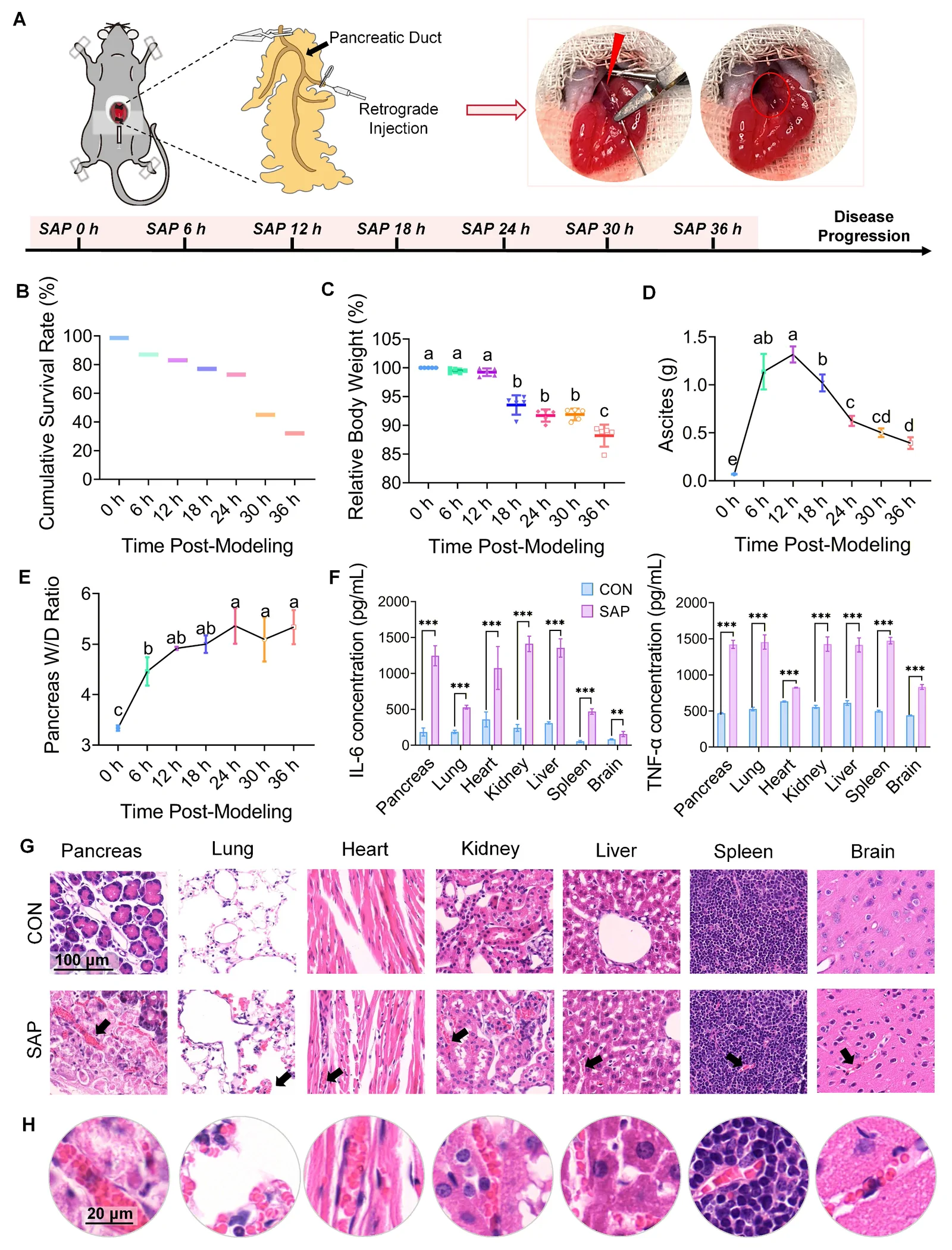
Pathological development of various organs in a mouse model of SAP. A) Diagram of SAP model development and experimental schedule. Red arrowhead denotes transient biliopancreatic duct clarity after successful cannulation; red circle denotes acute pancreatic tissue ischemic necrosis after infusion. B–E) Temporal dynamics of SAP: B) Cumulative survival rate of mice. C) Relative weight of mice. D) Ascites of mice. E) Pancreas W/D ratio. All values in C-F are presented as mean ± SD (n = 5 mice). Groups that do not have the same letter are significantly different (P < 0.05; Ordinary one-way ANOVA followed by Tukey’s HSD test). F) Levels of IL-6 and TNF-α in various organs of control (CON) and SAP 12 h groups (n = 5 mice). Asterisks denote significance: *P < 0.05, **P < 0.01, ***P < 0.001 (unpaired, two-tailed t-test or Welch's t-test). G) H&E-stained sections of several organs of CON and SAP 12 h groups. Tissues were perfused with PBS before collection to eliminate the presence of free-flowing blood. Erythrocyte aggregates (black arrows) are confirmed on the magnified image shown in the panel (H) (scale bar: 100 μm). H) 800× view of the same arrow-marked areas in (G). The typical biconcave shape of erythrocytes is evident inside the vessels. Scale bar: 20 μm.

In this study, we developed a mouse SAP model (Figure 1A). The survival rate was reduced to 73% at 24 h and further decreased to 32% by 36 h (Figure 1B). There were many manifestations such as weight loss (e.g., from 99.2% to 93.6% of the initial value between 12 h and 18 h; Figure 1C), massive abdominal fluid build-up (reaching a maximum of 1.3 g at 12 h; Figure 1D), and extreme pancreatic edema (Figure 1E). Moreover, SAP stimulated a serious systemic inflammatory response with an extreme increase in interleukin-6 (IL-6) and tumor necrosis factor-α (TNF-α) in major organs at 12 hours post-induction (Figure 1F).

H&E-stained sections revealed widespread erythrocyte aggregation in various organs (Figure 1G, black arrows; Figure 1H). Prior to tissue collection, we perfused the organs with PBS to remove free-flowing blood from the vasculature. We observed the erythrocyte aggregates in blood vessels in the SAP group. It means trapped red blood cells reflected vascular stasis or micro-thrombosis rather than residual circulating blood. These findings support the viewpoint that microcirculatory dysfunction and compromised vascular integrity are key contributors to the development of systemic injury in SAP.

### Development of 3D vascular imaging pipeline to analyze multi-organ vascular changes in SAP

**Figure 2:**
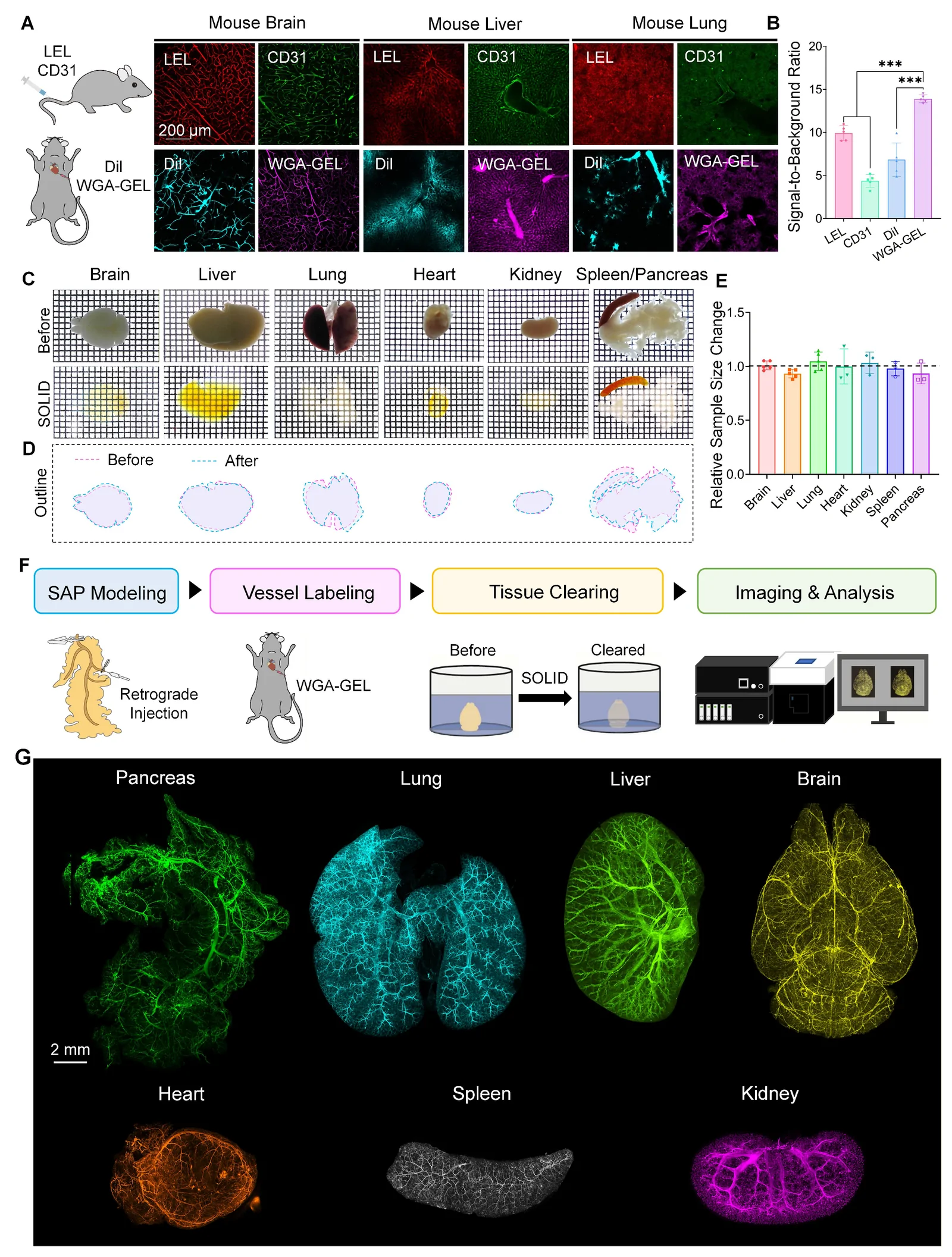
Systematic evaluation of vascular labeling and tissue clearing strategies for high-resolution 3D imaging of mouse organs. A) Maximum intensity projections (MIPs) of brain, liver, and lung vasculature labeled with four different vascular labeling methods: LEL, anti-CD31 (both injected via tail vein), DiI, WGA-GEL (both administered by cardiac perfusion). B) Quantitative analysis of cerebrovascular signal-to-background ratio (SBR) for the four labeling methods shown in (A). All values are presented as mean ± SD (n = 5 mice). *P < 0.05, **P < 0.01, ***P < 0.001 (Ordinary one-way ANOVA followed by Tukey’s HSD test). C) Transparency of different organs achieved by SOLID clearing method, with grid background (1.44 mm × 1.44 mm) for reference. The pancreas and spleen, which are anatomically contiguous in the mouse, were photographed together in a single image to retain their spatial relationship. D) Schematic of organ morphology quantification via outline tracing before and after clearing. E) Quantification of morphological changes in mouse organs post-clearing. All values are presented as mean ± SD (n = 5 mice for brain, liver, and lung; n = 3 mice for heart, kidneys, spleen, and pancreas). F) Experimental workflow, including SAP modeling, vessel labeling, tissue clearing, imaging & analysis. G) Representative 3D reconstructed vascular images of multiple organs using WGA-GEL labeling combined with SOLID clearing. Scale bar: 2 mm.

Given the potential central role of vascular injury in SAP progression, accurate and detailed 3D evaluation of microvascular alterations is essential. We first established a vascular labeling technique based on intracardiac perfusion of wheat germ agglutinin combined with gelatin (WGA-GEL). For comparison and benchmarking, conventional strategies, such as intravenous lycopersicon esculentum (tomato) lectin (LEL) or anti-CD31 antibody, and intracardiac DiI perfusion, were evaluated (Figure 2A). Quantitative analysis showed that the WGA-GEL method had a markedly higher signal-to-background ratio in cerebrovascular labeling compared to LEL, CD31, or DiI (Figure 2B). Notably, the WGA-GEL protocol achieved specific and uniform vascular labeling across multiple organs, including the brain, liver, and lung.

Next, we compared five tissue-clearing methods, including CUBIC, PACT, uDISCO, PEGASOS, and SOLID. SOLID offered the most favorable combination of high clearing efficacy and low structural distortion, and was consequently selected as the best performer for preserving the original shape of all seven organs (Figures 2C–E, Figures S1A–B). To enable direct transparency comparison while preserving anatomical context, we imaged the adjacent pancreas and spleen together in a single bright-field image (Figure 2C, S1A). In order to assess whether different clearing methods have any influence on vascular structural integrity, we compared the ratios of vessel diameter (cleared/before), branch point counts (cleared/before), and vascular volume fraction (VVF) (cleared/before) in brain sections before and after clearing. Quantification showed that the SOLID method was the most efficient in preserving the vessel diameter and branching structure and showed the smallest change in VVF (Figures S2A–C).

We established a powerful method by combining WGA-GEL labeling, SOLID clearing with light-sheet microscopy (Figure 2F) and applied it to mouse SAP model. This method delivered high-fidelity 3D reconstructions of the vascular network of seven organs, including pancreas, lung, heart, kidney, liver, spleen, and brain (Figure 2G).

### 3D imaging of the spatiotemporal progression of multi-organ vascular injury in SAP

**Figure 3:**
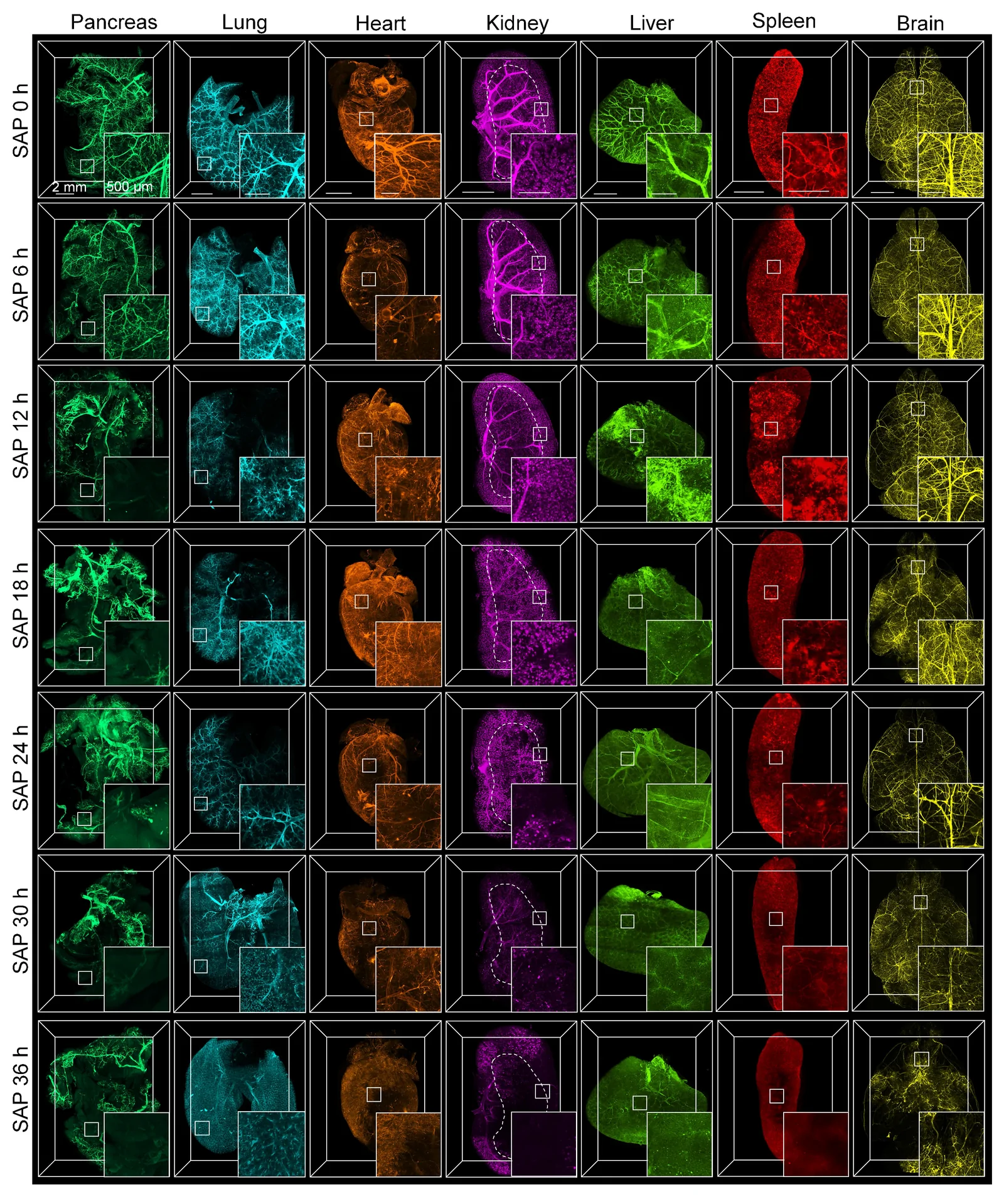
The 3D visualization of vascular integrity of seven organs in a mouse model of SAP. Pancreas, lung, heart, kidney, liver, spleen, and brain were collected and analyzed at different time points post- SAP induction (0, 6, 12, 18, 24, 30, and 36 h). Overall views of the whole organs (scale bar: 2 mm) with detailed images (scale bar: 500 μm) of the vascular structures.

**Figure 4:**
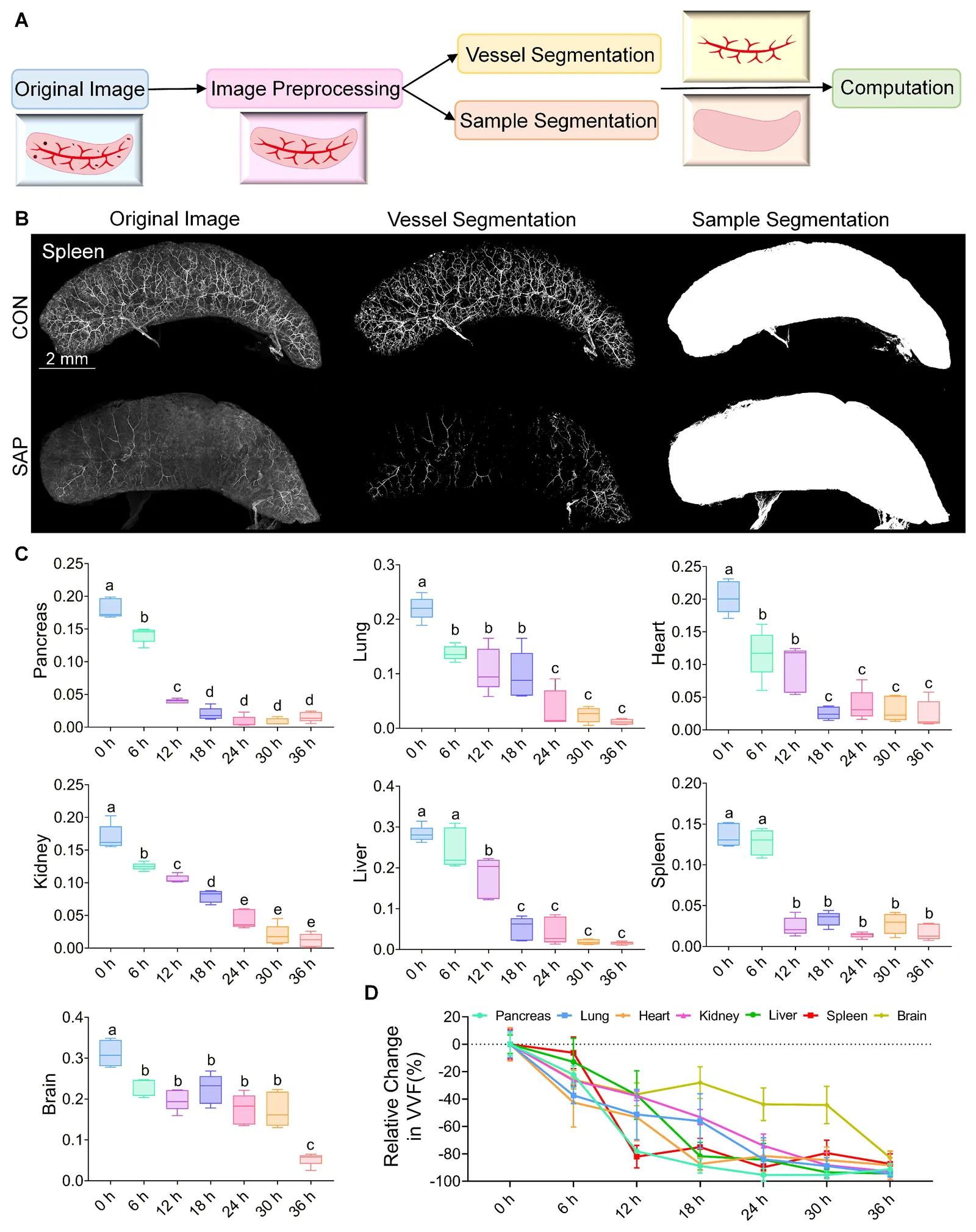
Quantitative profiling of vascular remodeling in multiple organs during SAP progression. A) Workflow for VVF quantification. B) Representative images of VVF calculation in the spleen from CON and SAP groups. Scale bar: 2 mm. C) VVF was measured in multiple organs at 0, 6, 12, 18, 24, 30, and 36 h after SAP induction. All values are presented as mean ± SD (n = 5 mice). Means with various letters are significantly different (P < 0.05; Ordinary one-way ANOVA with Tukey’s HSD post-hoc test or Welch's ANOVA with Dunnett’s T3 post hoc test). D) Time-course changes in relative VVF (normalized to CON) across organs during the 0–36 h period following SAP induction.

By applying the 3D imaging pipeline established in the previous section, we tracked the evolution of multi-organ vascular networks during SAP development (0–36 h) (Figure 3). The quantification of VVF is shown in Figure 4. By systematically acquiring and comparing 3D fluorescence images at different time points, it was visually illustrated that SAP-induced systemic vascular injury evolved asynchronously, with diverse spatiotemporal patterns across different organs.

The high-resolution 3D images demonstrated that the pancreas was the earliest and most severely affected organ, showing reduced perfusion at 6 h, and persisted hypoperfusion since 12 h. The lung had an ongoing deterioration of the vascular system, such as, a substantial decrease in perfusion could be observed at 6 h, and it further developed into a large-scale decrease in perfusion at 24 h. Similarly, cardiac perfusion declined progressively, and a slight decrease occurred at 6 h, but a significant decrease occurred at 18. Vascular injury of the kidney was minor at 6 h but the condition deteriorated severely after 12 h. For the liver and spleen, the perfusion level was good within 6 h, but suddenly decreased at 12 h, and then maintained hypoperfusion afterwards. It is worth noting that we did not observe obvious lesion in the cerebral vasculature at 12 h, however, after 18 h, the injury became obvious, and finally resulted in significant decline in perfusion during the end stage (30 to 36 h).

### Quantitative profiling of vascular injury reveals organ-specific patterns during SAP progression

We further employed our high-resolution 3D vascular imaging system to perform a quantitative analysis of vascular networks of multiple organs with the progression of SAP. Measurements of VVF are shown in Figure 4A and 4B. Analysis across time points produced organ-specific VVF trends (Figure 4C) showing significant spatiotemporal heterogeneity in vascular injury.

The pancreas was the first lesion site and showed a significant decrease in VVF within the first 6 h (from 18% to 14%), followed by a rapid decline to 4% at 12 h; it subsequently became severely hypoperfused. The lung showed progressive vascular deterioration as the VVF dropped to 14% by 6 h, and further decreased to 4% at 24 h, and 1% at 36 h. The heart VVF declined from 20% at 0 h to 12% by 6 h and further decreased to 3% at 18 h, and remained at 2–4%. The kidney VVF declined almost linearly with time between 0 and 36 h, declining from 17% to 1%. The liver and spleen were relatively stable at 6 h, but the liver dropped sharply to 5% at 18 h and the spleen showed significant decreases in VVF by 12 h, declining to 2%. Among all organs, the brain had the lowest rate of VVF reduction, remaining largely preserved until the late stage of disease, when VVF decreased to 5% at 36 h.

We normalized the VVF data of organs and compared organ-specific perfusion trajectories, so as to explore shared temporal patterns (Figure 4D). This result showed several distinct phases: from 0 to 6 h, the heart and lung VVF declined markedly fast. Between 6 and 18 h, the greatest decreases in VVF were recorded in the spleen (peak decline between 6 and 12 h) and the liver (peak decline between 12 and 18 h). From 18 to 30 h, the VVF of lung and kidney continued to decrease gradually. During 30-36 h, brain VVF declined sharply.

### Therapeutic efficacy of early heparin sodium administration in improving survival and preserving vascular network integrity in SAP

**Figure 5:**
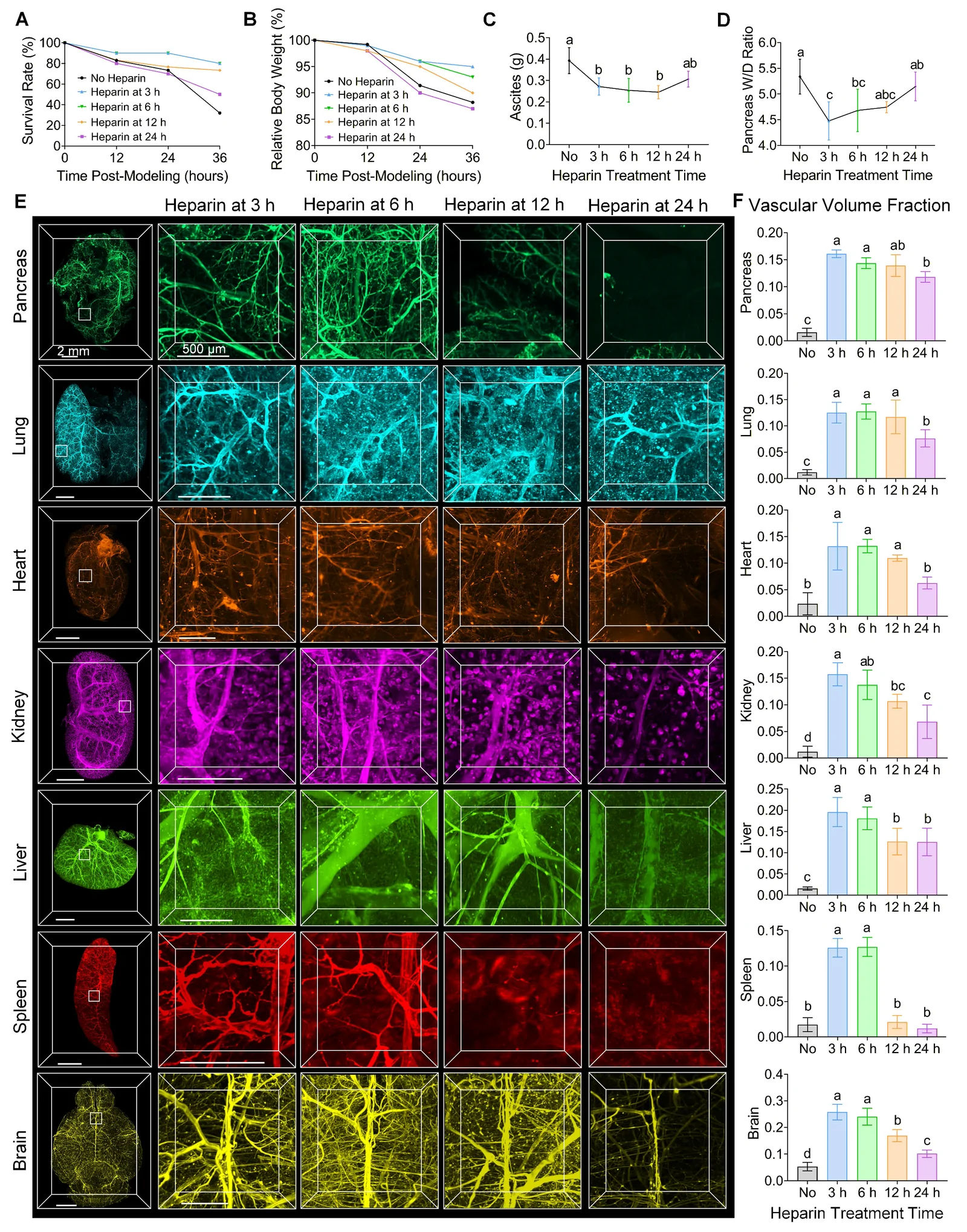
Timing-dependent therapeutic efficacy of heparin sodium in SAP. A) Cumulative survival rates in untreated and heparin sodium-treated groups. For simplicity, “heparin” is used in all figures to denote heparin sodium. B) Body weight changes over time. C) Ascites volume at 36 h after SAP induction. D) Pancreatic edema at 36 h. Data (C, D) are mean ± SD (n = 5). E) Representative 3D reconstructed vascular images of multiple organs from SAP mice treated with heparin at 3, 6, 12, and 24 h. Scale bar: 2 mm (overview), and 0.5 mm for the enlarged insets. F) VVF values compared among experimental groups. All values are presented as mean ± SD (n = 5 mice). Means with various letters are significantly different (P < 0.05, Ordinary one-way ANOVA with Tukey’s HSD post-hoc test or Welch's ANOVA with Dunnett’s T3 post hoc test); shared letters denote no statistical difference.

Next, we assessed the effect of heparin sodium administered at different time points after SAP induction. The therapeutic effect exhibited strong time-dependent profile. Early administration of heparin sodium obviously improved survival in the SAP model. When compared to the untreated group (a 32% survival rate), early treatment within 12 h post-induction increased survival to 70%-80% at 36 h (Figure 5A). Early treatment also alleviated body weight loss (Figure 5B), reduced ascites (Figure 5C) and pancreatic edema (Figure 5D). Delayed administration (24 h) was less effective.

To determine whether heparin sodium was able to protect vascular integrity, we performed 3D vascular imaging of multiple organs. Vascular images showed that early intervention (3, 6, or 12 h) mitigated SAP-induced vascular structural damage and preserved vascular network integrity (Figure 5E, Figure S3, Movies S1-S7). Quantitative analysis of VVF also supported these findings (Figure 5F). When given within 12 h, heparin sodium preserved vascular network integrity in the pancreas, heart, kidney, lung, brain, and liver. In the spleen, significant improvement was noticeable only at a narrow time window of 3 to 6 h.

Due to progressive kidney vascular injury, we further examined the glomeruli using a 3D quantitative analysis pipeline (Figure S4A). The use of 3D imaging and shape analysis allowed us to detect and quantify intact kidney vasculature and glomeruli (Figure S4B). The number of intact glomeruli decreased over time in almost a linear manner as SAP developed (Figure S4C), which was similar to the decrease in kidney VVF. Administration of heparin sodium significantly preserved the number of intact glomeruli and prevented microstructural loss. Taken together, these results indicate that early heparin sodium treatment maintained both renal vascular integrity and glomerular microarchitecture.

As an additional control, we performed dual-vessel labeling in heparin-treated mice to exclude any confounding effects of heparin on WGA distribution or VVF quantification. Heparin sodium was given 6 h after SAP induction. At the 36 h endpoint, 30 minutes prior to cardiac perfusion, CD31 antibody was injected via the tail vein to label perfused vessels in vivo. The mice were transcardially perfused with WGA-GEL. Tissues were cleared using the SOLID method and imaged by light-sheet microscopy. As shown in the representative 3D images of the brain and kidney, there was great colocalization of WGA-GEL (green) and CD31 (red), and the merged channels (yellow) confirmed consistent labeling of vascular structures (Figure S5A–D). Brain VVF values obtained with two labeling methods were similar (Figure S5E). The kidney VVF and glomerular counts also showed no meaningful discrepancy between the two methods (Figure S5F). These findings suggest that heparin sodium treatment did not affect WGA-GEL staining or its vascular distribution, supporting the validity of WGA-GEL labeling for structural measure of vascular integrity in this therapeutic context.

### Histological validation of endothelial protection by heparin sodium

**Figure 6:**
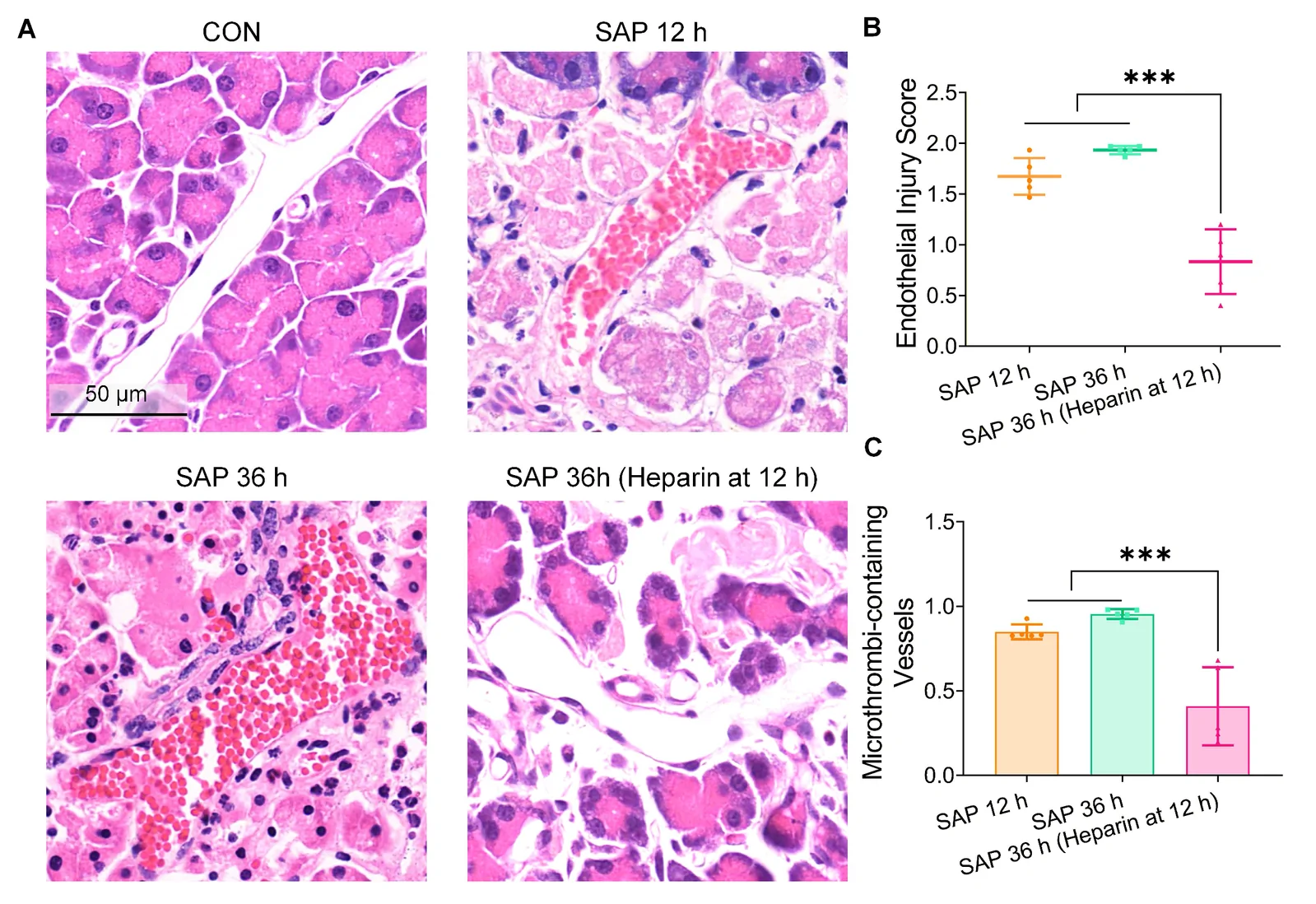
Histological evidence of endothelial injury and the protective effects of heparin sodium in SAP. A) Pancreatic sections stained with H&E in CON, SAP 12 h, SAP 36 h, and heparin sodium treated (heparin at 12 h, collected at 36 h) groups. Scale bar: 50 μm. B) Endothelial injury scores in SAP 12 h, SAP 36 h, and heparin sodium-treated groups. Scoring: 0, no damage; 1, mild injury; 2, severe injury. Ten randomly selected fields were scored per mouse. C) Proportion of vessels with erythrocyte aggregates or microthrombi in the SAP 12 h, SAP 36 h, and heparin sodium-treated groups. Ten randomly chosen fields were assessed per mouse, and the values were averaged to obtain a single mean proportion for each animal. All values are presented as mean ± SD (n = 5 mice). *P < 0.05, ** P < 0.01, *** P < 0.001 (one-way ANOVA followed by Tukey’s HSD test).

To assess endothelial damage and microthrombosis directly, pancreatic slices of control mice, SAP mice at 12 h and 36 h post-induction, and heparin sodium-treated SAP mice (heparin sodium administered at 12 h, tissues collected at 36 h) were stained with H&E. As shown in Figure 6A, there was significant endothelial injury in the SAP groups at both 12 and 36 h, which included swelling, detachment, and rupture of the vessel walls. The heparin sodium-treated group exhibited preserved endothelial integrity with slight changes in morphology.

Evaluation of endothelial injury with a scoring scale (0: no damage; 1: mild damage; 2: severe damage) showed that SAP 12 h and 36 h groups had comparable average scores, as compared to a markedly lower score in the heparin sodium-treated group (Figure 6B). We also calculated the percentage of vessels which had erythrocyte aggregates or microthrombi, and found that heparin sodium treated group showed significantly lower proportion than untreated groups (ten random visual fields were assessed per mouse) (Figure 6C). These data indicate that SAP is associated with endothelial damage and microvascular thrombosis, and early heparin sodium treatment can reduce these pathological changes.

## Discussion

A powerful combination of vascular labeling, tissue optical clearing and light-sheet imaging was applied to quantify multi-organ vascular injury in SAP. We show significant spatiotemporal variation in multi-organ vascular injury and that heparin sodium is effective only within a narrow early therapeutic window.

In this study, WGA-GEL was used to achieve uniform labeling of the vasculature of multiple organs. This perfusion strategy is similar to recently developed gelatin-based labeling methods, such as the VALID technology (DiI and hydrogel) 46, 47 and the Ultralabel pipeline (lysine-dextran/gelatin hydrogel) 48; however, VALID has poor compatibility with organic solvent–based clearing methods, and Ultralabel shows variable labeling performance across different organs. In addition, double labeling with WGA and Evans blue has been used to segment the cerebrovascular network, WGA alone is less effective for labeling large vessels 33. By contrast, the WGA–GEL method used in our study has extremely strong fluorescence signal, and great optical clearing compatibility, and uniform labeling ability to the vasculature of various organs. For tissue optical clearing, we chose the SOLID protocol because it provides the best balance between high transparency and minimal structural distortion.

The reduction of VVF that we have measured in the pancreas and kidney is also in accordance with previous reports of gradual deterioration of the vascular integrity in SAP 49. It is worth noting that the 3D quantitative analysis of the various organs in this study further revealed that there was significant heterogeneity in the timing and extent of vascular injury in different organs: the pancreas was the first to undergo a sharp vascular collapse, the kidney showed a continuous linear decline, and the brain perfusion was rapidly lost at the end stage, suggesting that SAP induced a sequential rather than synchronous systemic vascular injury process. It has been shown in the rat SAP model that the tight junction protein ZO-1 is significantly decreased in intestinal epithelial barrier as the disease progresses 50, which may be viewed as a molecular correlate of the structural disruption we have observed. And experimental findings demonstrating a correlation between disease severity and abnormal coagulation 51, which provides a mechanistic context for the extensive vascular injury recorded in our work.

Heparin sodium may exert its protective effects through anticoagulant, antithrombotic, and anti-inflammatory mechanisms, which may reverse SAP-associated hypercoagulability and hemoconcentration 52. The protective effect was highly time sensitive: when administered within 12 h it significantly improved vascular perfusion while delayed administration was ineffective. The clinical evidence suggests that intervention at an early stage with low molecular weight heparin is associated with reduced disease severity, pancreatic necrosis, mortality, organ failure, and thrombotic complications 53. All these results justify the importance of early heparin administration as a critical strategy. Nevertheless, heparin does not permeate the blood–brain barrier (BBB) well, which restricts central nervous system protection, and the oscillatory splenic vasomotor responses suggest that anticoagulation alone may not completely rehabilitate the immune organ function 54. Therefore, a combined therapy that contains both the anticoagulant and immunomodulatory properties may be required to achieve optimal protection.

While tools such as Vesselucida 360 and VesSAP offer detailed 3D vascular reconstruction, their computational cost is prohibitive for our large, multi-organ datasets. We therefore developed VVF, a simplified metric that capitalizes on the high signal-to-noise ratio of WGA-GEL imaging to rapidly quantify structural vascular patency at the organ level. VVF measures the fraction of tracer-accessible vascular volume and thus reflects patency rather than real-time perfusion. It cannot distinguish obstructed, collapsed, or thrombosed vessels, nor separate vasoconstriction from endothelial loss. Accordingly, functional confirmation with methods such as microbead perfusion, Doppler imaging, or hypoxia markers remains necessary to fully validate restored tissue perfusion.

The taurocholate-induced SAP model, although valuable for mechanistic insight, does not fully reflect human disease etiology, coagulation physiology, or progression. We did not evaluate clinical safety parameters such as optimal dosing or bleeding risk, nor did we account for inter-patient variability. Our findings on the therapeutic window and efficacy are therefore preclinical, and validation in large animal models to include a careful assessment of hemorrhagic risk will be essential before early heparin intervention can be considered for clinical use in SAP.

## Conclusions

This study establishes a comprehensive imaging approach for systemic 3D vascular visualization. It allows accurate assessment of the spatiotemporal dynamics of vascular remodeling in SAP and evaluates the efficacy of therapeutic strategies of early vascular-protective intervention. Using this approach, we found that multiple organ vascular injury in SAP did not occur simultaneously, but presented an orderly temporal and spatial cascade: the pancreas was the primary site of the first serious vascular collapse; the lung, heart and kidney showed progressive hypoperfusion; the liver and spleen deteriorated sharply after a period of relative stability; cerebral perfusion showed a sharp loss at the end stage. This organ-specific sequential injury model reveals the dynamic process of systemic vascular dysfunction in SAP. Our findings establish systemic vascular injuries as a central driver of multi-organ dysfunction in SAP. Administration of heparin sodium in a preclinical SAP mouse model within 12 h after model induction improved vascular network integrity and reduced microvascular injury. This work provides novel pathophysiological insight into SAP and establishes a critical need for early vascular protection to slow disease progression. It also defines a clear therapeutic window and offers an integrated research platform for future translational investigations of early vascular-protective therapies in SAP.

## Methods

*Animals:* All animals used in this study were male C57BL/6J mice (6–8 weeks old) obtained from Liaoning Changsheng Biotechnology Co., Ltd. and kept in the specific-pathogen-free (SPF) animal facility at Wuhan National Laboratory for Optoelectronics. Water and food were available ad libitum, and the light/dark cycle of 12 hours was kept throughout the study. All the animal procedures were performed in compliance with Experimental Animal Management Ordinance of Hubei Province, P. R. China, and were approved by the Institutional Animal Care and Use Committee of Huazhong University of Science and Technology ([2024] IACUC Number: 5284).

*Tissue Preparation:* The mice received deep anesthesia via intraperitoneal injection of ketamine (100 mg/kg) and xylazine (20 mg/kg) and were then transcardially perfused with 30 mL of 0.01 M phosphate-buffered saline (PBS) and 30 mL of 4% paraformaldehyde (PFA). The pancreas, lung, heart, kidney, liver, spleen, and brain were harvested for vascular imaging. Both kidneys were dissected from the mice. The same anatomically defined liver lobe was sampled from the animals. All tissues were kept in 4% PFA at 4 °C overnight, and then washed with PBS for 3 h, and stored in PBS until the subsequent steps of tissue clearing and imaging.

Table S1 provides a complete list of all reagents, drugs, dyes, and antibodies employed in this work.

*SAP Model Establishment:* Male C57BL/6J mice (20–25 g, 6–8 weeks) were fasted overnight with free access to water. The anesthesia was induced and maintained with 1.5% isoflurane delivered by means of a precision vaporizer. They were put onto a thermally controlled surgical table, which was set at 37 ± 0.5 °C. The midline of the abdomen was surgically cut, 1-1.5 cm in length, after the skin of the abdominal area was aseptic preparation. It was necessary to carefully exteriorize and retract the biliopancreatic duct and make sure that all important structures around were identified and protected precisely. The hepatic portal region was temporarily occluded by using a microvascular clip. A micro-infusion system was connected to a 30-gauge needle, which was inserted into the biliopancreatic duct through the duodenal wall and fixed. A 5% sodium taurocholate solution (50 μL per 100 g body weight) was infused retrograde over 2 minutes and lasted another 5 minutes. When the clip and needle were removed, the laparotomy was closed in layers. Fluid support was provided by giving physiological saline (25 mL/kg) subcutaneously. During the anesthetic recovery process, animals were kept on a thermoregulated warming pad and given appropriate analgesia until fully awake. The confirmation of pancreatic edema, hemorrhage, and necrotic patches during visual inspection helped to confirm the diagnosis of successful induction of SAP.

*Measurement of Ascites:* Ascites was quantified using a cotton ball absorption technique. In brief, after anesthesia, the abdominal cavity was exposed via a midline incision. Pre-weighed sterile cotton balls were repeatedly used to absorb the fluid until no further exudation was detected. The fully soaked cotton balls were weighed; the dry cotton ball weight was subtracted to obtain the net ascites weight.

*Assessment of Pancreatic Edema:* Pancreatic water content, a hallmark of acute pancreatitis, was evaluated by the wet-to-dry weight ratio (W/D). Immediately after euthanasia, the pancreas was excised, blotted gently to remove surface fluid, and weighed to obtain the wet weight (W). The samples were desiccated in the oven at 60 °C. All samples were reweighed at regular intervals until the mass stabilized after about 48 h; this value was taken as the dry weight (D). The W/D ratio was then calculated to quantify tissue edema.

*Detection of Cytokines:* Supernatants of tissue homogenates were collected, and the IL-6 and TNF-α levels were measured with commercial ELISA kits as per the manufacturer guidelines. The specific steps included the incubation of antibodies, the reaction of enzyme-conjugate, and development of the substrate color. It was measured as optical density at 450 nm and the concentrations of the cytokines were then determined.

*Hematoxylin–eosin (H&E) Staining:* Samples were fixed in 4% PFA for over 48 h, then dehydrated in an ethanol series, embedded in paraffin, and sectioned at 4–6 μm. H&E staining was done on deparaffinized and rehydrated sections. Following dehydration and clearing, the sections were mounted with neutral resin. Whole-slide digital images were then obtained using a slide scanner.

*LEL and anti-CD31 Labeling*: After deep anesthesia, to dilate the tail vein and ease the injection procedure, the tail was placed in 40 °C water for 30 s. Once vasodilation was evident, LEL or anti-CD31 antibody (200 μL per 20 g body weight in saline) was injected through the dilated tail vein.

*DiI and WGA-GEL Labeling:* Mice were deeply anesthetized as described above. After blood was cleared by transcardial perfusion with 0.01 M PBS, the heart was infused with 10–15 mL of the selected labeling solution at a flow rate of 1–2 mL/min. For labeling with DiI, perfusion was performed using the working solution, which contained DiI at 0.4 μg/mL. The successful perfusion was indicated by a slight purple coloration of the extremities.

For WGA-GEL labeling, the labeling solution was composed of 0.1 mg WGA powder dissolved in 15 mL of a warm 2% (w/v) aqueous gelatin solution. Perfusion was carried out under warm conditions to maintain the liquidity of the gelatin. The carcass was subsequently stored at 4 °C overnight to allow gelatin solidification within the vasculature. WGA binds to the endothelial glycocalyx, while the hydrogel mixture simultaneously fills the vascular lumen. This combined labeling method strongly marks patent, well-perfused vessels. Vessels that are narrowed, collapsed, or blocked by microthrombi exhibit reduced fluid flow and consequently decreased delivery of the labeling agent. Therefore, the resulting fluorescent signal reflects both the presence of endothelial cells and the patency of vessels at the time of perfusion.

*Tissue Clearing:* SOLID 45: Tissue clearing was performed as previously described, using a two-step process involving gradient delipidation/dehydration and refractive index matching. Specifically, the samples were incubated with an array of 1,2-hexanediol solutions, with 30%, 50%, 70%, and 90% (v/v), each supplemented with 2% (v/v) N-butyldiethanolamine. Finally, the tissue was immersed in HxD-TBN, a mixture composed of 90% (v/v) 1,2-hexanediol, 10% (v/v) tert-butanol, and 2% (v/v) N-butyldiethanolamine. With the whole mouse brain as a standard, the samples were agitated at 30 °C for total of five sequential incubation steps: the first and last each lasted 1 day, while the three intervening steps were each carried out for 0.5 days. Thereafter, the specimens were transferred to BBPN solution—a mixture prepared with 75% benzyl benzoate, 20% PEGMMA500, and 5% N-butyldiethanolamine (all % are v/v)—and held there until optical transparency was achieved.

CUBIC 55: The samples were delipidated using a CUBIC-L formulation composed of 10% (w/w) N-butyldiethanolamine and 10% (w/w) Triton X-100. The immersion proceeded at 37 °C for 4–7 days, after which a 12 h PBS wash was applied. For refractive index matching, the specimens were then transferred to CUBIC-R medium containing antipyrine at 45% (w/w) and nicotinamide at 30% (w/w), and held at 37 °C for an additional 2–4 days. Gentle shaking was maintained throughout the entire protocol.

PACT 56, 57: After fixation in 4% PFA, the tissue sections were immersed at 4 °C overnight in the A4P0 hydrogel monomer formulation that was made up of 4% acrylamide and 0.25% VA-044 photoinitiator in PBS. Unpolymerized hydrogel was washed off using a brief rinse of PBS followed by placing the hybrids into 50 mL tubes filled with a clearing buffer composed of 8% SDS in PBS. The delipidation was obtained by shaking the tubes at 37 °C during 2–5 days. The optical clearing was performed by transferring the samples with a refractive index matching solution, 70% (w/v) sorbitol in distilled water; and continuing with the immersion until total transparency was obtained.

uDISCO 58: Fixed tissues were dehydrated in a stepwise manner using ascending concentrations of tert-butanol (tB) (30%, 50%, 70%, 80%, 90%, 96%, and 100%, v/v), with each dehydration step carried out at 35 °C under continuous agitation. Subsequently, refractive index matching was achieved with BABB-D4, which was prepared by first mixing benzyl alcohol and benzyl benzoate (1:2, v/v) to obtain BABB, then adding diphenyl ether at a BABB:DPE ratio of 4:1, and finally supplementing with 0.4% (v/v) DL-α-tocopherol.

PEGASOS 59: After decolorization in 25% (v/v) Quadrol for 2 days, the samples were dehydrated stepwise through 30%, 50%, and 70% (v/v) tB (12 h per step), each containing 3% (w/v) Quadrol. They were then immersed in tB-PEG (70% tB/27% PEGMMA500/3% Quadrol; w/v for Quadrol, v/v for other liquids) for 2 days, and cleared to transparency in BB-PEG (75% benzyl benzoate/25% PEGMMA500/3% Quadrol). All the operations were done at 37°C with continuous gentle stirring.

*Heparin Sodium Treatment Protocol:* Powdered heparin sodium (150 IU/mg) was suspended in sterile physiological saline to obtain a working concentration of 12 IU/mL. The solution was mixed by vortexing to achieve complete dissolution, filtered with a 0.22 μm filter, divided into aliquots, and stored at 4 °C out of the light. Before use, the solution was warmed to room temperature. Heparin sodium (60 IU/kg) was injected via the tail vein. This dose was selected based on its established efficacy–safety profile: it is highly effective in normalizing microcirculatory disturbances and inhibiting inflammatory reactions when applied in a range of 50–100 IU/kg without causing serious hemorrhagic complications 60. In all figures, “heparin” is used in short form to refer to heparin sodium in order to be clear.

*Optical Imaging:* Bright-field overviews were acquired with a Sony digital camera (model WW119533). Fluorescence micrographs of vascular labeling in tissue slices were recorded on a Zeiss LSM 710 confocal microscope running Zen 2011 SP2 (v8.0.0.273). Wide-field scans used a 5× objective lens, numerical aperture (NA) = 0.25, and working distance (WD) = 12.5 mm, while cellular details were resolved with a 10× objective lens (NA = 0.50; WD = 2.0 mm). Serial optical sections (Z-stacks) were collected for three-dimensional reconstruction. For intact, optically cleared organs (cleared by the SOLID method), whole-mount 3D vasculature was visualized on a LiTone XL light-sheet fluorescence microscope. Tissues were illuminated with a thin light-sheet and imaged through a 4× objective lens (NA = 0.28; WD = 20 mm). Dual-side views were simultaneously recorded and computationally fused, producing isotropic, uniformly resolved datasets. Representative images shown in the main figures were selected as follows: all five replicates per organ and time point were first inspected to confirm image quality and absence of artifacts. One replicate was then chosen as a typical example that captures the shared group characteristics.

*Relative signal-to-background ratio (SBR) measurement:* An effort was made to measure the fluorescence preservation; relative SBR values were determined according to the following procedure. Maximum intensity projections (MIP) were first computed from image stacks collected at 50 μm beneath the tissue surface. Vascular structures were then identified by applying the “Threshold” function in Fiji to the MIP images, and the mean intensity of the threshold regions was taken as the signal. As a reference, an adjacent area devoid of vessels was selected, and its mean intensity was recorded as the background value. The SBR was computed by dividing the mean signal intensity by the mean background intensity.

*Quantification of Relative Sample Size Change:* Bright field images before and after tissue optical clearing were used to evaluate tissue size alterations. Using the Fiji (v1.53, https://fiji.sc/) “Polygon Selections” tool, the outline of each sample was manually traced on top-view images to measure its area. The relative change in tissue size was then expressed as the percentage variation in area between the pre- and post-clearing states.

*Measurement of Vascular Volume Fraction (VVF):* VVF is described as the proportion of labeled vascular structures in the tissue or organ. This measure indicates the relative quantity of patent, perfused vessels and can be used as an indicator of vascular network integrity and patency. Semi-automatic extraction of vascular signals was performed on tissue images by applying an intensity threshold (Figure 4A). Binary images were generated and VVF was then calculated in MATLAB by determining the volume ratio of the vasculature to the total sample volume. The equation below was used to determine the VVF values:



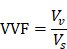



In this case, V*_v_* is the vascular volume and V*_s_* is the total sample volume. At each time point, VVF was calculated from five mice; standard deviations were derived from these five biological replicates. This metric in question was created in order to offer the quantitative measure of the overall vascular perfusion capacity in each organ.

*Statistical Analysis:* All quantitative data are mean ± SD, and the number of samples (n) is given in the figure legends. The Shapiro–Wilk test was applied to determine whether normality exists. If the data met normality and homogeneity of variances, ordinary one-way ANOVA with Tukey's HSD post hoc test was used. Otherwise, Welch's ANOVA with Dunnett's T3 post hoc test was adopted instead. Independent samples t-test (or paired t-test for paired samples) was used to compare two groups if the data had normal distributions and homogeneity of variances; otherwise, Welch's t-test (for independent samples) and the Wilcoxon signed-rank test (for paired samples) were used. To denote statistical significance in figures: for experiments comparing two or three groups, significance is indicated directly with asterisks (*P < 0.05, **P < 0.01, ***P < 0.001); when comparing more than three groups (i.e., four or more groups), a lowercase letter-coding scheme is applied, where any two groups not sharing a common lowercase letter are considered significantly different (P < 0.05).

## Supplementary Material

Supplementary figures and table, movies.

## Data Availability

The data that support the findings of this study are available from the corresponding author upon reasonable request.

## Abbreviations

3D: three-dimensional
SAP: severe acute pancreatitis
MODS: multiple organ dysfunction syndrome
AP: acute pancreatitis
WGA: wheat germ agglutinin
GEL: gelatin
SOLID: suppressing tissue distortion based on synchronized dehydration/ delipidation treatment with 1,2-hexanediol mixtures
LEL: lycopersicon esculentum (Tomato) lectin
CD31: cluster of differentiation 31
CUBIC: clear unobstructed brain imaging cocktails and computational analysis
PACT: passive clear lipid-exchanged anatomically rigid, imaging/ immunostaining-compatible tissue hydrogel technique
uDISCO: ultimate dimensional imaging of solvent-cleared organs
PEGASOS: polyethylene glycol methacrylate-associated solvent system
VVF: vascular volume fraction
