# Supplementary material for: 3D vascular mapping reveals multi-organ injury following severe acute pancreatitis
Source: Theranostics. 2026 Aug 24;16(15):8844–59. doi: 10.7150/thno.141299 (PMC13549375; doi:10.7150/thno.141299)
Supplement: Supplementary file 1 [file thnov16p8844s1.zip › Supporting Information.pdf]

Supporting Information for

3D vascular mapping reveals multi-organ injury following severe acute pancreatitis

Xiaomei Liu†, Jingtan Zhu†, Qiao Shi†, Jianyi Xu, Zhang Liu, Yujia Kang, Kunxing Liu, Yuening He, Qihang Yang, Tingting Yu, and Dan Zhu**

X. Liu, J. Xu, Z. Liu, Y. Kang, K. Liu, Y. He, Q. Yang, T. Yu, D. Zhu

MOE Key Laboratory for Biomedical Photonics, Wuhan National Laboratory for Optoelectronics-Advanced Biomedical Imaging Facility, Huazhong University of Science and Technology, Wuhan, Hubei 430074, China

Optics Valley Laboratory, Wuhan, Hubei 430074, China

Correspondence to: yutingting@hust.edu.cn; dawnzh@mail.hust.edu.cn

J. Zhu

Center for Medical Genetics, School of Life Sciences, MOE Key Laboratory of Rare Pediatric Diseases, Hunan Key Laboratory of Animal Models for Human Diseases, Central South University, Changsha, Hunan 410031, China

Q. Shi

Department of Hepatobiliary and Pancreatic Surgery, Renmin Hospital of Wuhan University, Wuhan, Hubei 430060, China

†Xiaomei Liu, Jingtan Zhu and Qiao Shi contributed equally to this work.

This word file includes:

Figure S1 to Figure S5

Figure S1. Evaluation of clearing efficacy and morphological preservation of different clearing methods across various organs.

Figure S2. Assessment of fine vascular architecture preservation before and after clearing with different methods.

Figure S3. Temporal analysis of systemic vasculature during heparin sodium treatment.

Figure S4. Quantification of renal glomerular changes during SAP progression and the impact of heparin sodium treatment.

Figure S5. Validation of WGA-GEL vascular labeling under heparin sodium treatment.

Table S1

Table S1. List of the all reagents, drugs, dyes, and antibodies used in this study.

Additional supporting files accompanying this paper are listed below:

Movie S1. 3D visualization of the pancreas at 0 h and 24 h after SAP induction, and at 36 h after heparin administration given 3 h post-induction.

Movie S2. 3D visualization of the lung at 0 h and 24 h after SAP induction, and at 36 h after heparin administration given 3 h post-induction.

Movie S3. 3D visualization of the heart at 0 h and 24 h after SAP induction, and at 36 h after heparin administration given 3 h post-induction.

Movie S4. 3D visualization of the kidney at 0 h and 24 h after SAP induction, and at 36 h after heparin administration given 3 h post-induction.

Movie S5. 3D visualization of the liver at 0 h and 24 h after SAP induction, and at 36 h after heparin administration given 3 h post-induction.

Movie S6. 3D visualization of the spleen at 0 h and 24 h after SAP induction, and at 36 h after heparin administration given 3 h post-induction.

Movie S7. 3D visualization of the brain at 0 h and 24 h after SAP induction, and at 36 h after heparin administration given 3 h post-induction.

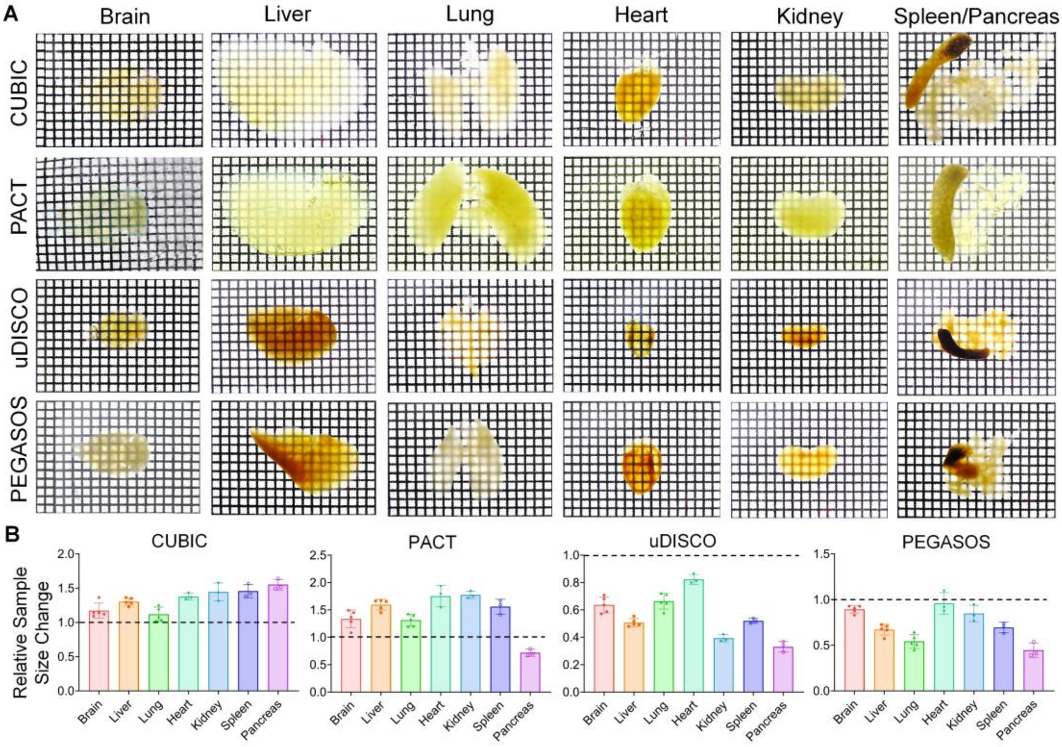

Figure S1. Evaluation of clearing efficacy and morphological preservation of different clearing methods across various organs. A) Transparency of different organs after clearing with CUBIC, PACT, uDISCO, and PEGASOS, with a grid background (1.44 mm × 1.44 mm) for reference. The pancreas and spleen were photographed together in a single image, due to their anatomical connection. B) Quantification of morphological changes (relative sample size change) in the indicated organs after clearing with each method. Data are mean ± SD (n = 5 mice for brain, liver, and lung; n = 3 mice for heart, kidney, spleen, and pancreas).

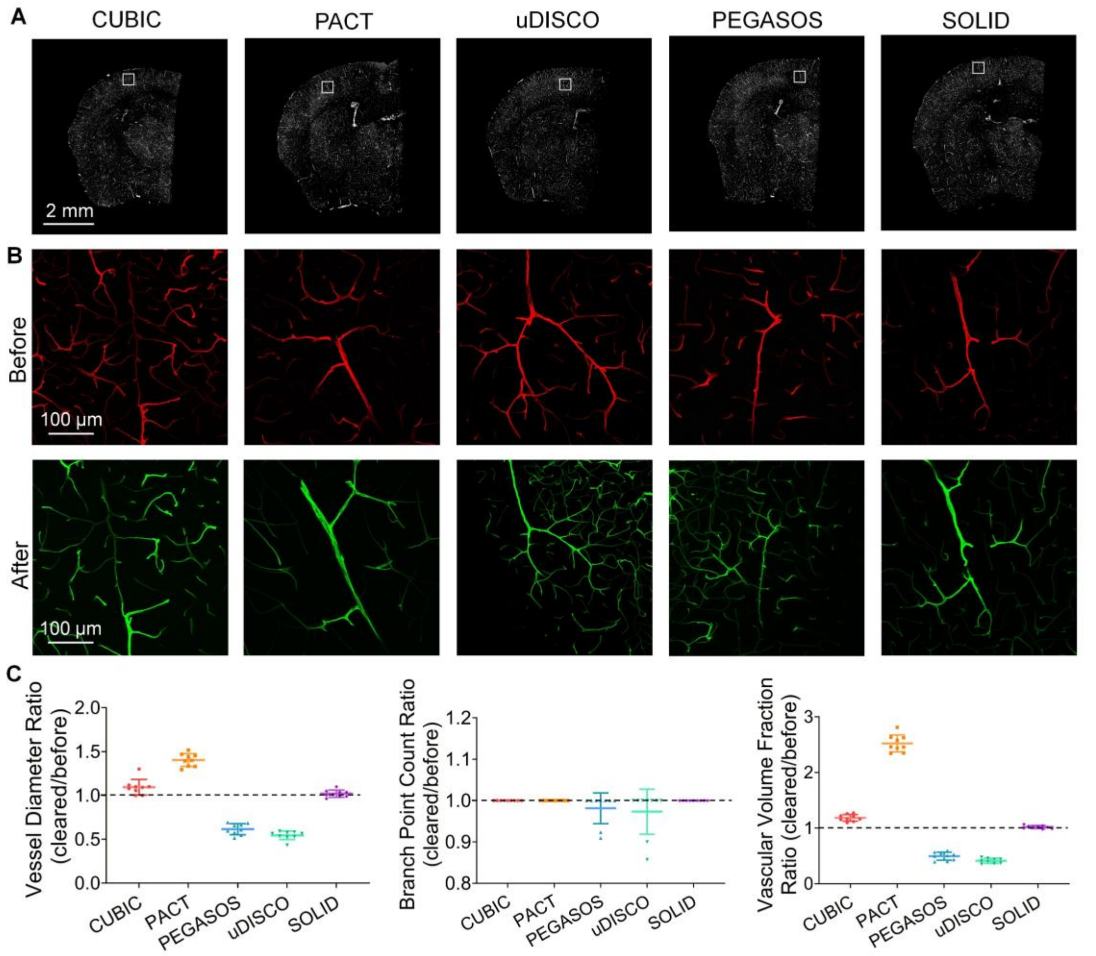

Figure S2. Assessment of fine vascular architecture preservation before and after clearing with different methods. A) Confocal tile-scan MAP images (10×, zoom 0.6) of mouse hemibrain slices before clearing, showing the anatomical locations sampled for high-magnification analysis in (B). White solid boxes indicate selected regions. Scale bar: 2 mm. B) Confocal maximum intensity projections (30 μm z-stack, 20× objective) of vascular structures before (red) and after (green) clearing with the indicated protocols, acquired from the boxed areas in (A). Scale bar: 100 μm. C) Quantification of vessel diameter ratio (cleared/before), branch point count ratio (cleared/before), and vascular volume fraction ratio (cleared/before). Data are mean ± SD (n = 9 ROIs from 3 mice for each method).

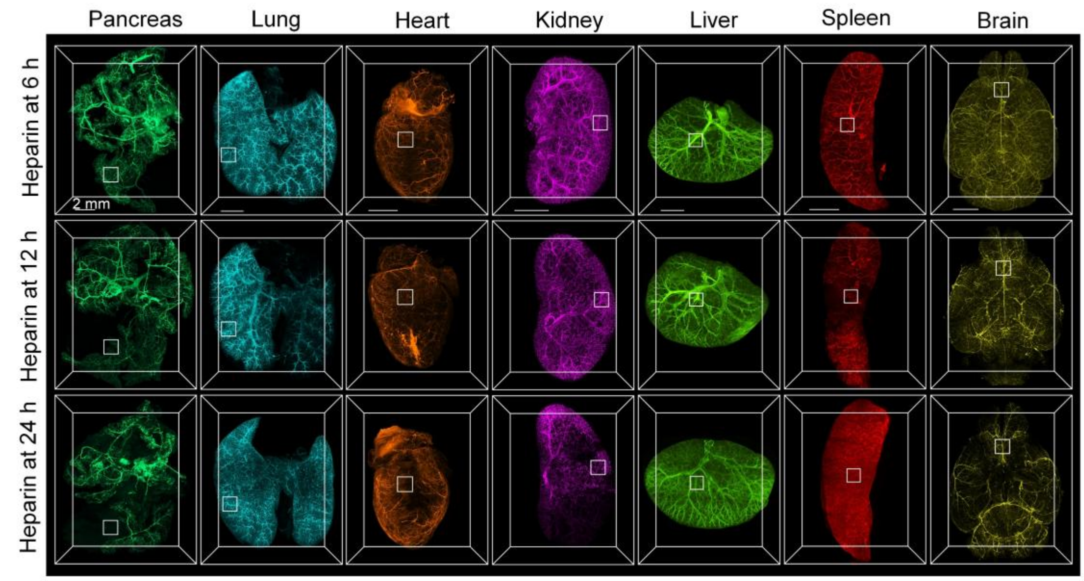

Figure S3. Temporal analysis of systemic vasculature during heparin sodium treatment. Representative 3D reconstructed images of systemic vasculature from SAP mice treated with heparin sodium at 6, 12, and 24 h post-modeling, demonstrating the progressive therapeutic benefits on vascular perfusion. These observations visually support the quantitative rescue effects quantified in Figure 5E. Scale bar: 2 mm.

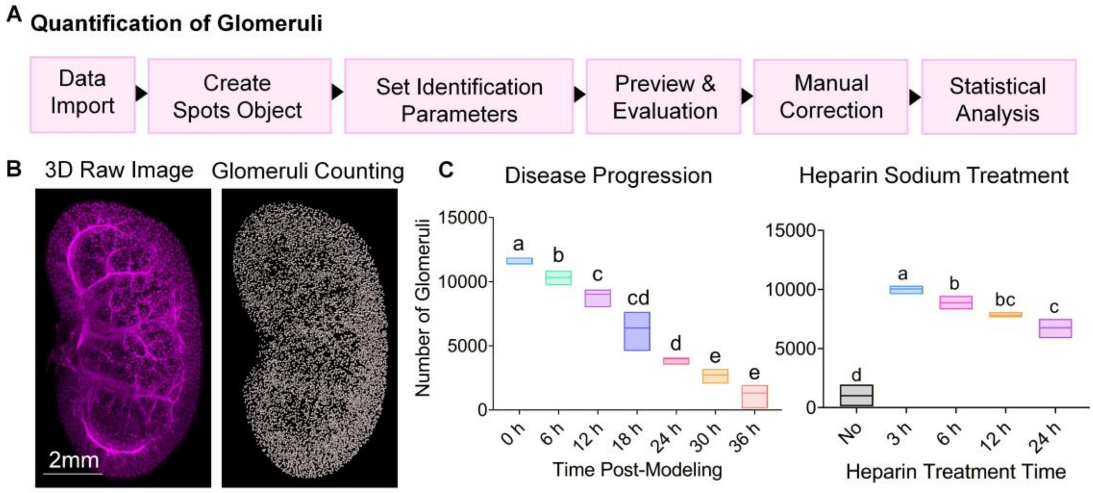

Figure S4. Quantification of renal glomerular changes during SAP progression and the impact of heparin sodium treatment. A) Schematic workflow for the quantitative analysis of glomeruli in kidney tissue. B) Representative images showing the glomerular identification process. Left: Original 3D fluorescence image of renal vasculature. Right: Corresponding image with detected glomeruli highlighted. Scale bar: 2 mm. C) Glomerular counts across experimental groups, including the SAP time course (0–36 hours) and heparin sodium treatment administered at multiple time points. All values are expressed as mean ± SD (n = 5 mice per group). Means bearing distinct lowercase letters differ significantly ($P < 0.05$; Ordinary one-way ANOVA with Tukey's HSD post-hoc test or Welch's ANOVA with Dunnett T3 post hoc test). Groups that share a common letter are not statistically different.

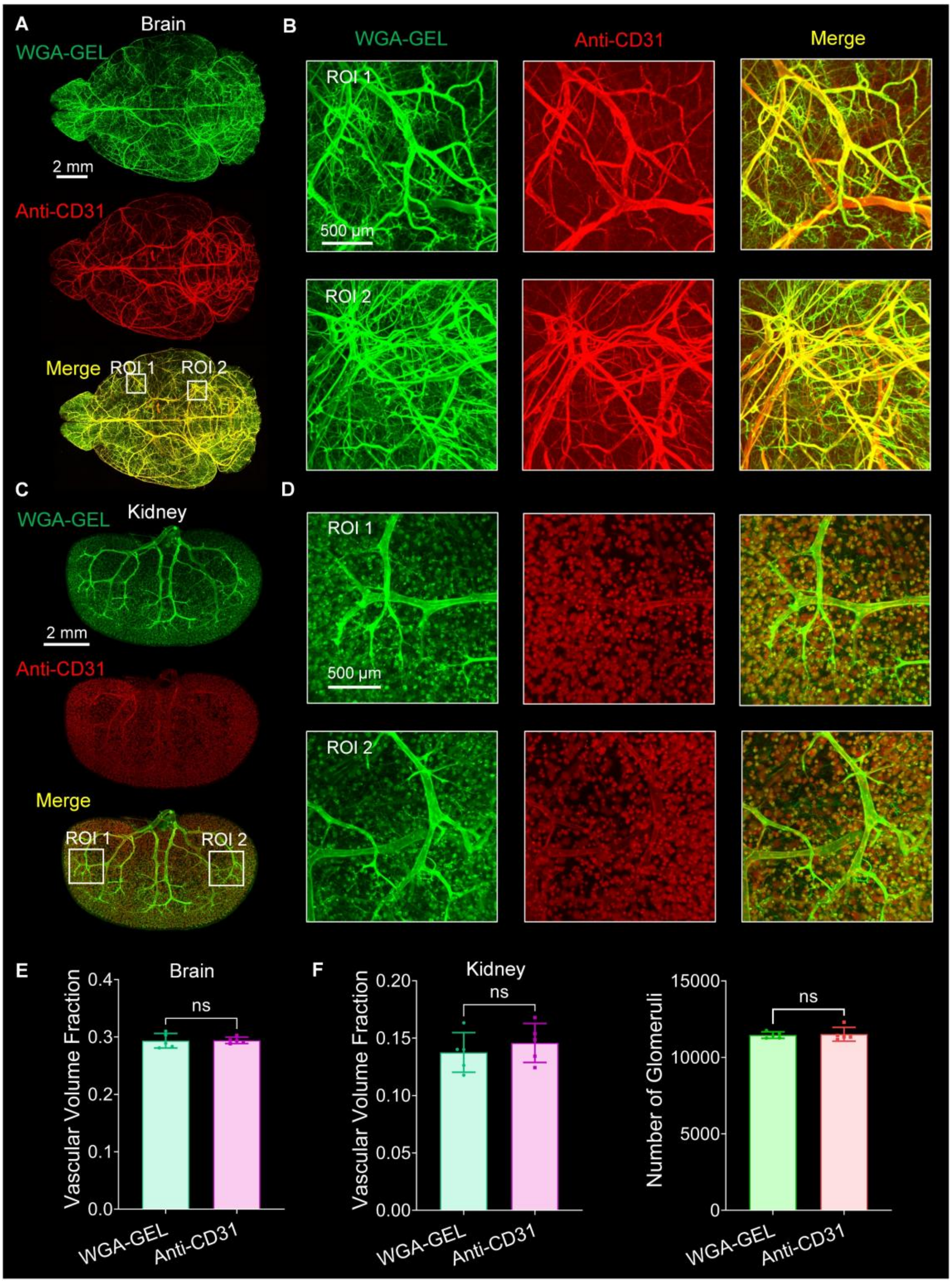

Figure S5. Validation of WGA-GEL vascular labeling under heparin sodium treatment. A) Representative 3D reconstructed image of mouse brain vasculature after dual labeling with WGA-GEL (green) and CD31 (red) in heparin-treated mice at 36 h post-SAP induction. Scale bar: 2 mm. B) MIPs of the magnified views of ROI1 and ROI2 indicated in (A), showing detailed colocalization of WGA-GEL and CD31. Scale bar: 0.5 mm. C) Representative 3D reconstructed image of mouse kidney vasculature after dual labeling with WGA-GEL (green) and CD31 (red) under the same experimental conditions. Scale bar: 2 mm. D) MIPs of the magnified views of ROI1 and ROI2 indicated in (C), showing detailed colocalization of WGA-GEL and CD31. Scale bar: 0.5 mm. E)

Quantification of VVF in brain labeled with WGA-GEL versus CD31. Values are shown as mean ± SD (n = 5 mice for each group). ns, non-significant (Ordinary one-way ANOVA with Tukey's HSD post-hoc test or Welch's ANOVA with Dunnett T3 post hoc test). F) Comparison of VVF and glomerular counts in kidney stained with WGA-GEL and with CD31. Data are expressed as mean ± SD (n = 5 mice for each group). ns, no significant difference (paired, two-tailed t-test).

Table S1 List of the reagents, drugs used in this study.

Reagent Name	Company	Catalog Number
Phosphate buffer solution	Sigma-Aldrich	P3813
Paraformaldehyde	Sigma-Aldrich	158127
Ketamine	China Animal Husbandry Group Co., Ltd.	100761663
Xylazine	Sigma-Aldrich	X1251
Pork skin gelatin	Sigma-Aldrich	V900863
Ethanol	Sinopharm Chemical Reagent Co., Ltd.	10009218
1,2-hexanediol	Macklin	H916855
N-butyldiethanolamine	Tokyo Chemical Industry	B0725
Tert-butanol	Sigma-Aldrich	360538
Benzyl benzoate	Sigma-Aldrich	W213802
Polyethylene glycol methacrylate 500	Sigma-Aldrich	447943
Triton X-100	Sinopharm Chemical Reagent Co., Ltd.	30188928
Antipyrine	Tokyo Chemical Industry	D1876
Nicotinamide	Tokyo Chemical Industry	N0078
Acrylamide	Sigma-Aldrich	A108468
VA-044 photoinitiator	Wako Chemicals	011-19365
Sodium Dodecyl Sulfate	Sinopharm Chemical Reagent Co., Ltd.	30166428
Histodenz	Sigma-Aldrich	I134719
Tween-20	Bio-Rad	1706531
Sodium azide	Sigma-Aldrich	S2002
Benzyl alcohol	Sigma-Aldrich	W213705
Diphenyl ether	Sigma-Aldrich	W366706
DL-α-tocopherol	Alfa Aesar	A17039
Quadrol	Tokyo Chemical Industry	T0781
Sodium Taurocholate	Sigma-Aldrich	T4009
Heparin sodium	Sinopharm Chemical Reagent Co., Ltd.	H8060
Xylene	Sinopharm Chemical Reagent Co., Ltd.	10023418
Hematoxylin	Sinopharm Chemical Reagent Co., Ltd.	H8070
Eosin	Sinopharm Chemical Reagent Co., Ltd.	HE1001
IL-6 ELISA Kit	ABclonal Technology Co., Ltd.	RK00028
TNF-α ELISA Kit	ABclonal Technology Co., Ltd.	RK00072
LEL- Dylight 594	Vector Laboratories	DL-1177
Anti-CD31 Antibody-Alexa Fluor 647	BioLegend	102416
DiI	Aladdin	D131225
WGA-Alexa Fluor 555	Thermo Fisher Scientific	W32464

Movie S1. 3D visualization of the pancreas at 0 h and 24 h after SAP induction, and at 36 h after heparin administration given 3 h post-induction.

Movie S2. 3D visualization of the lung at 0 h and 24 h after SAP induction, and at 36 h after heparin administration given 3 h post-induction.

Movie S3. 3D visualization of the heart at 0 h and 24 h after SAP induction, and at 36 h after heparin administration given 3 h post-induction.

Movie S4. 3D visualization of the kidney at 0 h and 24 h after SAP induction, and at 36 h after heparin administration given 3 h post-induction.

Movie S5. 3D visualization of the liver at 0 h and 24 h after SAP induction, and at 36 h after heparin administration given 3 h post-induction.

Movie S6. 3D visualization of the spleen at 0 h and 24 h after SAP induction, and at 36 h after heparin administration given 3 h post-induction.

Movie S7. 3D visualization of the brain at 0 h and 24 h after SAP induction, and at 36 h after heparin administration given 3 h post-induction.
